# Connecting Higher-Order Separation Logic to a First-Order Outside World

**DOI:** 10.1007/978-3-030-44914-8_16

**Published:** 2020-04-18

**Authors:** William Mansky, Wolf Honoré, Andrew W. Appel

**Affiliations:** ETH Zurich, Zurich, Switzerland; 9 University of Illinois at Chicago, Chicago, IL USA; 10 Yale University, New Haven, CT USA; 11 Princeton University, Princeton, NJ USA

**Keywords:** formal verification, verifying communication, modular verification, interaction trees, VST, CertiKOS

## Abstract

Separation logic is a useful tool for proving the correctness of programs that manipulate memory, especially when the model of memory includes higher-order state: Step-indexing, predicates in the heap, and higher-order ghost state have been used to reason about function pointers, data structure invariants, and complex concurrency patterns. On the other hand, the behavior of system features (e.g., operating systems) and the external world (e.g., communication between components) is usually specified using first-order formalisms. In principle, the soundness theorem of a separation logic is its interface with first-order theorems, but the soundness theorem may implicitly make assumptions about how other components are specified, limiting its use. In this paper, we show how to extend the higher-order separation logic of the Verified Software Toolchain to interface with a first-order verified operating system, in this case CertiKOS, that mediates its interaction with the outside world. The resulting system allows us to prove the correctness of C programs in separation logic based on the semantics of system calls implemented in CertiKOS. It also demonstrates that the combination of interaction trees + CompCert memories serves well as a *lingua franca* to interface and compose two quite different styles of program verification.

## 1 Introduction

Separation logic allows us to verify programs by stating pre- and postconditions that describe the memory usage of a program. Modern variants include reasoning principles for shared-memory concurrency, invariants of locks and shared data structures, function pointers, rely-guarantee-style reasoning, and various other interesting features of programming languages. To support these features, the "memory" that is the subject of their assertions is not just a map from addresses to values, but something more complex: it may contain "predicates in the heap" to allow reasoning about invariants attached to dynamically allocated objects such as semaphores, it may be step-indexed to allow higher-order assertions, and it may contain various forms of ghost state describing resources that exist only for the purposes of verification. The soundness proof of the logic then relates these decorated heaps to the simple address-map view of memory used in the semantics of the target language.

This works well as long as every piece of the system is verified with respect to decorated heaps, but what if we have multiple verification tools, some of which provide correctness results in terms of undecorated memory (or, still worse, memory with a different set of decorations)? To take advantage of the correctness theorem of a function verified with one of these tools, we will need to translate our decorated memory into an undecorated one, demonstrate that it meets the function's undecorated precondition, and then take the memory output by the function and use it to reconstruct a decorated memory. In this paper, we demonstrate a technique to do exactly that, allowing higher-order separation logics (in this instance, the Verified Software Toolchain) to take advantage of correctness proofs generated by other tools (in this case, the CertiKOS verified operating system). This allows us to remove the separation-logic-level specifications of system calls from our trusted computing base, instead relying on the operating system's proofs of its own calls. In particular, we are interested in functions that do more than just manipulate memory (which is separation logic's specialty)—they communicate with the outside world, which may not know anything about program memory or higher-order state.

```
int main(void) {
  unsigned int n, d; char c;
  n=0;
  c=getchar();
  while (n<1000) {
    d = ((unsigned)c)-(unsigned)'0';
    if (d>=10) break;
    n+=d;
    print_int(n);
    putchar('\n');
    c=getchar();
  }
  return 0;
}
```

Fig. 1: A simple communicating program

Consider the program in Figure 1. It repeatedly reads a digit from the console, adds it to the sum of the digits seen so far, and prints the current sum to the console. Although this is a very simple program, it is not a natural fit for separation-logic-based verification tools, which model the behavior of C programs in terms of computation and memory rather than I/O. Several approaches have been suggested for reasoning about I/O in separation logic, for instance by Penninckx et al. [18] and Koh et al. [13]. Using the latter approach, we might specify the behavior of getchar with the Hoare triple $\{\mathsf{ITree}(r \leftarrow \mathsf{read};;\ k\ r)\}$ x = getchar() $\{\mathsf{ITree}(k\ \mathrm{x})\}$, relating the function call to an external read event: the program before the call to getchar must have permission to perform a sequence of operations beginning with a read, and after the call it has permission to perform the remaining operations (with values that may depend upon the received value). By adding these specifications as axioms to VST's separation logic, we can use standard separation logic techniques to prove the correctness of programs such as the one above. But when we compile and run this C program, putchar and getchar are not axiomatized functions; they are system calls provided by the operating system, which may have an effect on kernel memory, user memory, and of course the console itself. If we prove a specification of this C program using the separation logic rules for putchar and getchar, what does that tell us about the behavior of the program when it runs? For programs without external calls, we can answer this question with the soundness proof of the logic. To extend this soundness proof to programs with external calls, we must relate the pre- and postconditions of the external calls to both the semantics of C and their implementations in the operating system.

In this paper, we describe a modular approach to proving soundness of a verification system for communicating programs, including the following elements:

- An extension of VST with support for generic ghost state.
- A generic mechanism for reasoning about external communication in a higher-order separation logic, built on top of ghost state.
- A technique for relating pre- and postconditions for external functions in higher-order separation logic to first-order specifications of the same functions in the verified operating system CertiKOS, with a general approach to "de-step-indexing" a certain class of step-indexed specifications.
- A new notion of correctness of the implementation of external communication, by relating user-level traces of external behavior to I/O operations inside the operating system.

The result is the first soundness proof of a separation logic that can be extended with first-order specifications of system calls. All proofs are formalized in the Coq proof assistant.

To understand the scope of our results, it is important to clarify exactly how much of CertiKOS we have brought into our proofs of correctness for C programs, and how much of a gap remains. The semantics on which we prove the soundness of our separation logic is the standard CompCert semantics of C, extended with the specifications of system calls provided by CertiKOS. Our model does not include the process by which CertiKOS switches from user mode to kernel mode when executing a system call, but rather assumes that CertiKOS implements this process so that the user cannot distinguish it from a normal function call. To prove this assertion rather than assuming it, we would need to transfer our soundness proof to the whole-system assembly-language semantics used by CertiKOS, and interface with not just CertiKOS's system call specifications but also its top-level correctness theorem. We discuss this last gap further in Section 7, but in summary, we prove that our client-side programs and OS-side system calls are correct, while *assuming* that CertiKOS correctly implements its transition between user mode and kernel mode.

The rest of the paper proceeds as follows. In Section 2, we describe generic ghost state in separation logic. In Section 3, we show how to encode the state of the outside world as ghost state that can only be changed through calls to external functions, allowing us to describe external communication in separation logic specifications. In Section 4, we use this approach to specify console I/O operations, and demonstrate the verification of a simple communicating program. In Sections 5 and 6, we describe the process of verifying the implementation of an external call, by first connecting its VST specification to a first-order specification on memory and then relating that "dry" specification to the functional specification of the same call in CertiKOS. This allows us to state our central theorem, which guarantees that programs verified in VST run correctly given the CertiKOS system call specifications. In Section 7, we address the relationship between user-level events and the actual communication performed by the OS. In Sections 8 and 9, we review related work and summarize our results.

## 2 Background: Ghost State in Separation Logic

### 2.1 Ghost Algebras

The fundamental insight behind ghost state is that if a mathematical object has the same basic properties as a separation logic heap, it can be injected into separation logic as a resource, even if it is not actually present in program memory. This insight was discovered independently by many people [4,3,19], and the "basic properties" required have been characterized in many ways: partial commutative monoids (PCMs), resource algebras, separation algebras, etc. They all include the idea that the ghost state must support an *operator*, often written as $\cdot$, for combining it in the same way heaps are combined by disjoint union, and they require that operator to have some of the properties of heap union (associativity, commutativity) but not all (for instance, it may be possible to combine two identical pieces of ghost state). Crucially, the operator $\cdot$ may be *partial*, so that the very existence of one piece of state means that another piece cannot possibly exist in the same program (just as ownership of one piece of the heap means that no other thread can hold the same piece). We follow Iris [11] in also including a *validity* predicate valid that marks out the elements of an algebra that represent well-formed ghost state.

Ghost state appears in the logic in a new kind of assertion, which we write as own, asserting that the current thread owns a certain ghost resource. In the assertion own $g\ a\ pp$, $g$ is an identifier (analogous to a location in the heap), $a$ is an element of the underlying algebra, and $pp$ is a predicate, allowing for a limited form of higher-order ghost state—for instance, we can store separation logic assertions in ghost state to implement global invariants. The key property of the own assertion is that separating conjunction on it corresponds to the $\cdot$ operator of the underlying algebra (see rule own_op in Figure 2). By defining different algebras with different operators, we can define different sharing protocols for the ghost state. For instance, if we only want to count the number of times some shared resource is used, the state may be a number and the operator

$$\textsf{own\_op}\ \frac{a1 \cdot a2 = a3}{\textsf{own}\ g\ a3\ pp \Leftrightarrow \textsf{own}\ g\ a1\ pp * \textsf{own}\ g\ a2\ pp}$$

$$\textsf{own\_update}\ \frac{\textsf{fp\_update}\ a\ b}{\textsf{own}\ g\ a\ pp \Rrightarrow \textsf{own}\ g\ b\ pp}$$

$$\textsf{consequence}\ \frac{P \Rrightarrow P' \qquad \{P'\}\ C\ \{Q'\} \qquad Q' \Rrightarrow Q}{\{P\}\ C\ \{Q\}}$$

Fig. 2: Key separation logic rules for ghost state may be addition; if we want to describe the pattern of sharing more precisely, as with ghost variables, the state may be a pair of the variable's value and a fraction of ownership, with a guarantee that two fractions are only compatible if they agree on the value. More complex sharing patterns correspond to more complicated join operations; for instance, Jung et al. [11] showed that any acyclic state machine can be encoded as ghost state, with the join operation computing the closest common successor of two states. The ghost state is not explicitly referenced by program instructions, but it can be modified at any time via a *frame-preserving update*: ghost state $a$ can be replaced with $b$ as long as any third party's ghost state $c$ that is consistent with $a$ is also consistent with $b$, formally expressed as $\textsf{fp\_update}\ a\ b \triangleq \forall c, a \cdot c \Rightarrow b \cdot c$, where we write $a \cdot b$ to mean $\exists d.\ a \cdot b = d$, i.e., $a$ and $b$ are compatible pieces of ghost state. This frame-preserving update is embedded into the logic using a *view-shift* operator $\Rrightarrow$, as shown in rule own_update of Figure 2.

```
           x = 0;
acquire(l); || acquire(l);
x++;        || x++;
release(l); || release(l);
```

Fig. 3: The increment example

Figure 3 shows the canonical example of a program where ghost state increases the verification power of separation logic. Using concurrent separation logic as originally presented by O'Hearn [17], we can prove that the value of `x` at the end of the program is at least 0, but we cannot prove that it is exactly 2. This limitation comes from the fact that we can associate an *invariant* with the lock `l`, but that invariant cannot express *progress* properties such as a change in the value of `x`. We can get around this limitation by adding ghost state that captures the contribution of each thread to `x`, and then use the invariant to ensure that the value of `x` is the sum of all contributions. (This approach is due to Ley-Wild and Nanevski [16].) We begin with ghost state that models the central operation of the program:

**Definition 1.** *The* sum *ghost algebra is the algebra* $(\mathbb{N}, +, \lambda n.\mathsf{True})$ *of natural numbers with addition, in which every number is a valid element.*

Intuitively, the lock invariant should remember every addition to `x`, while each individual thread only knows its own contribution. This is actually an instance of a very general pattern: the *reference* pattern, in which one party holds a complete and correct "reference" copy of some ghost state, and one or more other parties hold possibly incomplete "partial" copies. Because the reference copy must always be completely up to date, the partial copies cannot be modified without access to the reference copy. When all the partial copies are gathered together, they are guaranteed to accurately represent the state of the data structure. The reference ghost algebra is built as follows:

**Definition 2.** *Given a ghost algebra $G$, we define the* positive *ghost algebra on $G$, written $\mathsf{pos}(G)$, as an algebra whose carrier set is $(\Pi \times G) \cup \{\bot\}$, where $\Pi$ is a set of shares.*[4] *An element of $\mathsf{pos}(G)$ is* valid *if it has a nonempty share, and the operator $\cdot$ is defined such that $(\pi_1, a_1) \cdot (\pi_2, a_2) = (\pi_1 + \pi_2, a_1 \cdot a_2)$ and $x \cdot \bot = x$ for all $x$.*

The *positive* ghost algebra contains pairs of a nonempty share and an element of $G$, with join defined pointwise, representing partial ownership of an element of $G$. Total ownership of the element can be recovered by combining all of the pieces, obtaining a full share, and combining all of the $G$ elements accordingly.

**Definition 3.** *Given a ghost algebra $G$, let the* reference *ghost algebra on $G$, written $\mathsf{ref}(G)$, be the algebra $(\mathsf{pos}(G) \times (G \cup \bot), \cdot, \{(p, r) \mid r = \bot \vee p \sqsubseteq r\})$, where $(p_1, r) \cdot (p_2, \bot) = (p_1 \cdot p_2, r)$, and $p \sqsubseteq r \triangleq \exists q.\ p \cdot q = (\top, r)$.*

An element of the *reference* ghost algebra is a pair of a positive share of $G$ (partial element) and an optional reference element of $G$, where the reference element is unique and indivisible, and the partial element must be completable to the reference element if one exists. This ensures that when all the shares are gathered, i.e., when the partial element is $(\top, a)$, then it exactly matches the reference element, but no changes can be made to the partial element without the reference element present. To more clearly relate elements of this algebra to their intended meanings, we write $\mathsf{ref}\ r$ for the reference element $(\bot, r)$ and $\mathsf{part}\ s\ v$ for the partial element $((s, v), \bot)$.

Now we can formalize our intuition about what each party knows about the sum. We let the lock invariant for `l` be $\exists v.\ x \mapsto v * \mathsf{own}\ g\ (\mathsf{ref}\ v)$, and start each thread with a partial element $\mathsf{part}\ \frac{1}{2}\ 0$. When each thread acquires its lock and increments `x`, it also uses the own_update rule to increment its partial ghost state. At the end of the program, we can combine the two partial elements to obtain $\mathsf{part}\ \top\ 2$, which in combination with the lock invariant is sufficient to guarantee that the value of `x` is 2. This pattern can be used for a wide range of applications

[4] We use tree shares [1, Chapter 41] in the Coq proofs, but for simplicity of presentation in this paper we will use fractional shares: $\bot$ is the empty share, $\frac{1}{2}$ is a half share, and $\top$ is the full share.

by replacing the sum algebra with one appropriate to the application or data structure in question. We will also make use of it later to model the state of the external world as a separation logic resource.

### 2.2 Semantics of Ghost State

To support the use of ghost state in a separation logic, we need to make two main changes in the construction of the logic. First, we need to extend the underlying model of the logic with ghost state: rather than being predicates on the heap, our assertions are now predicates on the combination of heap and ghost state. Once ghost state exists in the model, we can give semantics to the $\mathsf{own}$ assertion.

Second, we need to change our definition of Hoare triples to allow for the possibility of frame-preserving updates to ghost state at any point in a program's execution. In a ghost-free separation logic, we might define Hoare triples with respect to an operational semantics for the language as follows:

$$[\![\{P\}\ c\ \{Q\}]\!] \triangleq \forall h, P(h) \Rightarrow (c, h) \rightarrow^* (\mathsf{done}, h') \Rightarrow Q(h')$$

where $(c, h) \rightarrow (c', h')$ means that the program $c$ executed with starting heap $h$ may take a step to a new program $c'$ with heap $h'$. For a step-indexed logic, it is more convenient to write this definition inductively:

**Definition 4 (Safety).** *A configuration* $(c, h)$ *is* safe for $n$ steps with postcondition $Q$ *if:*

- *$n$ is 0, or*
- *$c$ has terminated and $Q(h)$ holds to approximation (step-index) $n$, or*
- *$(c, h) \rightarrow (c', h')$ and $(c', h')$ is safe for $n - 1$ steps with $Q$.*

We can then define $\{P\}\ c\ \{Q\}$ (at step-index $n$) to mean that $\forall h.\ P(h) \Rightarrow (c, h)$ is safe for $n$ steps with $Q$.

Once we have added ghost state, our heap $h$ is now a pair $(h, g)$ of physical and ghost state, and between any two steps the ghost state may change. This leads us to a ghost-augmented version of safety.

**Definition 5 (Safety with Ghost State).** *A configuration $(c, h, g)$ is safe for $n$ steps with postcondition $Q$ if:*

- *$n$ is 0, or*
- *$c$ has terminated and $Q(h, g)$ holds to approximation $n$, or*
- *$(c, h) \rightarrow (c', h')$ and $\forall g_{\text{frame}}.\ g \cdot g_{\text{frame}} \Rightarrow \exists g'.\ (g' \cdot g_{\text{frame}} \wedge (c', h', g')$ is safe for $n - 1$ steps with $Q)$.*

The program must be able to continue executing under any $g_{\text{frame}}$ consistent with its current ghost state, but its choice of new ghost state $g'$ may depend on the frame. This quantifier alternation captures the essence of ghost state: the ghost state held by the program constrains any other ghost state held by the notional "rest of the system", and may be changed arbitrarily in any way that does not invalidate that other ghost state.

## 3 External State as Ghost State

An I/O-performing program modifies the state of the outside world. We would like to treat this external state as a kind of ghost state, since it is not in the program's memory and yet can be described by separation logic assertions. At the same time, we would emphatically *not* like to allow users to make arbitrary frame-preserving updates to external state: the external environment should have complete control of the external state, and the program should never be able to change it except by calling external functions. Furthermore, VST's semantic model (used to prove soundness) already includes an external state element[5], a black box of arbitrary type that is carried around by the program and passed to the environment at each external call, allowing the effects of external calls to be stateful without explicitly representing their state in program memory. While this external state is present in the operational semantics of VST, prior to the changes we describe it could not be referred to by separation logic assertions and was never instantiated with anything other than the singleton type `unit`. In this section, we describe how we combine ghost state with the built-in external state to make the external state visible in the separation logic.

Intuitively, external state is just another kind of shared resource, and we should be able to model it with a form of ghost state. However, one of the key features of ghost state is that programs can make arbitrary frame-preserving updates to it, while programs should never be able to modify external state. We can accomplish this using the reference ghost algebra of Section 2: the reference element $\mathsf{ref}\ a$ will be held by the external environment, while the program holds a partial element $\mathsf{part}\ \top\ a$. This ensures that the program cannot make any frame-preserving updates without the reference element, which is only available when the program passes control to the external environment via an external call. It then remains to choose the underlying algebra $G$ of the external state. Different applications may call for external state with different carrier sets and operations, but in the simplest case, the VST user will not want to split or combine the local copy of the external state[6]. In this case, they can pick a type $Z$ and make $G$ the *exclusive* ghost algebra for $Z$, which holds only an empty unit element and an indivisible ownership element, preventing the local copy from being divided. Then the user program holds an element $\mathsf{part}\ \top\ a$ that cannot be divided or modified, but only passed to the external environment, where $a : Z$ is the current value of the external state. We encapsulate the ghost state construction in an assertion $\mathsf{has\_ext}\ a \triangleq \mathsf{own}\ 0\ (\mathsf{part}\ \top\ a)$, where 0 is the identifier reserved for the external ghost state. Now, when verifying a program with external state, the user simply provides the starting state $a$, and receives in the precondition of the `main` function the assertion $\mathsf{has\_ext}\ a$, with no need to use or understand the ghost state mechanism.

[5] Appel et al. [1] call this the *external oracle*, but we refer to it as simply "external state" to avoid confusion with the environment oracles of CertiKOS.

[6] One example of a use case that benefits from nontrivial external state structure is a multithreaded web server in which different threads serve different clients simultaneously; in this case, each thread might have its own piece of the external state.

On the back end, we must still modify VST's semantics to connect the ghost state $a$ to the actual external state, and to prevent the "ghost steps" of the semantics from changing the external state. Recall from Section 2 that in order for a non-terminated configuration $(c, h, g)$ to be safe for a nonzero number of steps, it must be the case that $(c, h) \to (c', h')$ and $\forall g_{\text{frame}}.\ g \cdot g_{\text{frame}} \Rightarrow \exists g'.\ g' \cdot g_{\text{frame}} \wedge (c', h', g')$ is safe. To connect the external ghost state to a real external state $z$, we simply extend this definition to require that $g_{\text{frame}}$ include an element $(\bot, z)$ at identifier 0. This enforces the requirement that the value of the external ghost state always be the same as the value of the external state, and ensures that frame-preserving updates cannot change the value of the external state. Re-proving the separation logic rules of Verifiable C with this new definition of Hoare triple required only minor changes, since internal program steps never change the external ghost state.

When the semantics reaches an external call, the call is allowed to make arbitrary changes to the state consistent with its pre- and postcondition, including changing the value of the external ghost state (as well as the actual external state). We can use has_ext assertions in the pre- and postcondition of an external function to describe how that function affects the external state. For instance, we might give a console `write` function the "consuming-style" specification $\{\mathsf{has\_ext}(\mathsf{write}(v);;\ k)\}$ $\texttt{write}(v)$ $\{\mathsf{has\_ext}(k)\}$, stating that if before calling $\texttt{write}(v)$ the program has permission to write the value $v$ and then do the operations in $k$, then after the call it is left with permission to do $k$. (We could reverse the pre- and postcondition for a "trace-style" specification, in which the external state records the history of operations performed by the program instead of the future operations allowed.) In this paper, we use *interaction trees* [13] as a means of describing a collection of allowed traces of external events. Interaction trees can be thought of as "abstract traces with binding"; for instance, we can write $x \leftarrow \mathsf{read};;\mathsf{write}\ (x + 1);;k\ x$ to mean "read a value, call it $x$, write the value $x + 1$, and then continue to do the actions in $k$ using the same value of $x$."

In the end, we have a new assertion has_ext on external state that works in exactly the way we expect: it can hold external state of any type, it cannot be modified by user code, it can be freely modified by external calls, it always has exactly the same value as the external state already present in VST's semantics, and it exposes no ghost-state functionality to the user. If the user wants more fine-grained control over external state (for instance, to split it into pieces so multiple threads can make concurrent calls to external functions), they can define their own ghost algebra for the state and pass around part elements explicitly, but for the common case, has_ext provides seamless separation-logic reasoning about C programs that interact with an external environment.

## 4 Verifying C Programs with I/O in VST

Once we have separation logic specifications for external function calls, verifying a communicating program is no different from verifying any other program. We demonstrate this with the example program excerpted in Figure 1, shown in

$\{\mathsf{ITree}(\mathsf{write\_list}(\mathsf{decimal\_rep}'(i));;\ k)\}$

```
void print_intr(unsigned int i) {
  unsigned int q,r;
  if (i!=0) {
    q=i/10u;
    r=i%10u;
    print_intr(q);
    putchar(r+'0');
  }
}
```

$\{\mathsf{ITree}(k)\}$

$\{\mathsf{ITree}(\mathsf{write\_list}(\mathsf{decimal\_rep}(i));;\ k)\}$

```
void print_int(unsigned int i) {
  if (i==0)
    putchar('0');
  else print_intr(i);
}
```

$\{\mathsf{ITree}(k)\}$

$\{\mathsf{ITree}(c \leftarrow \mathsf{read};;\ \mathsf{main\_loop}(0, \mathsf{c}))\}$

```
int main(void) {
  unsigned int n, d; char c; n=0;
  c=getchar();
  while (n<1000) {
    d = ((unsigned)c)-
(unsigned)'0';
    if (d>=10) break;
    n+=d;
    print_int(n);
    putchar('\n');
    c=getchar();
  }
  return 0;
}
```

$\{\mathsf{ITree}(\mathsf{done})\}$

Fig. 4: A simple communicating program, with specifications for each function full in Figure 4. The print_intr function uses external calls to putchar to print the decimal representation of its argument, as long as that argument is nonzero; print_int handles the zero case as well. The main function repeatedly reads in digits using getchar and then prints the running total of the digits read so far. The ITree predicate is simply a wrapper around the has_ext predicate of the previous section (i.e., an assertion on the external ghost state), specialized to interaction trees on I/O operations. We can then write simple specifications for getchar and putchar, using interaction trees to represent external state:

$$\{\mathsf{ITree}(r \leftarrow \mathsf{read};;\ k\ r)\}\ x = \mathrm{getchar}()\ \{\mathsf{ITree}(k\ x)\}$$
$$\{\mathsf{ITree}(\mathsf{write}(x);;\ k)\}\ \mathrm{putchar}(x)\ \{\mathsf{ITree}(k)\}$$

Next, we annotate each function with separation logic pre- and postconditions; the program does not manipulate memory, so the specifications only describe the I/O behavior of each function. The effect of print_intr is to make a series of calls to putchar, printing the digits of the argument i as computed by the meta-level function decimal_rep′ (where $\mathsf{write\_list}([i_0; i_1; ...; i_n])$ is an abbreviation for the series of outputs $\mathsf{write}(i_0);;\ \mathsf{write}(i_1);;\ ...;;\ \mathsf{write}(i_n)$). When the value of i is 0, print_intr assumes that the number has been completely printed, so print_int adds a special case for 0 as the initial input. The specification for the main loop is a recursive sequence of read and write operations, taking the running total (which starts at 0) and the most recent input as arguments:

$$\begin{aligned}&\mathsf{main\_loop}(n,d) \triangleq \text{if } n < 1000\\ &\quad \text{then } \mathsf{write\_list}(\mathsf{decimal\_rep}(n+d));;c \leftarrow \mathsf{read};;\ \mathsf{main\_loop}(n+d,c) \text{ else } \mathsf{done}\end{aligned}$$

Using the specifications for putchar and getchar as axioms, we can easily prove the specifications of print_intr, print_int, and main. (The following sections show how we substantiate these axioms.)

$\{\mathsf{ITree}(\ell \leftarrow \mathsf{read\_list}(n);;\ k\ \ell) * \mathit{buf} \mapsto \_\}$
$x = \text{getchars}(\mathit{buf}, n)$
$\{\exists vs.\ \mathsf{length}(vs) = n \wedge x = n \wedge \mathsf{ITree}(k\ \ vs) * \mathit{buf} \mapsto vs\}$

$\{\mathsf{length}(vs) = n \wedge \mathsf{ITree}(\mathsf{write\_list}(vs);;\ k) * \mathit{buf} \mapsto vs\}$
$\text{putchars}(\mathit{buf}, n)$
$\{\mathsf{ITree}(k) * \mathit{buf} \mapsto vs\}$

Fig. 5: Separation logic specifications for I/O calls with memory

More complicated programs may manipulate memory as well as communicating, and we can easily combine the two. For instance, if we want to read or write several characters in a single call, the standard C idiom is to pass a buffer in memory as an argument. Figure 5 shows the specifications for functions putchars and getchars in this style, where each function takes as arguments a buffer to hold the input/output and a number indicating the size of the buffer[7]. The pre- and postconditions of these functions now involve both the external state and a standard points-to assertion for the buffer. (Note that $\ell \leftarrow \mathsf{read\_list}(n)$ is an abbreviation for the series of inputs $\ell_0 \leftarrow \mathsf{read};;\ \ell_1 \leftarrow \mathsf{read};;\ ...;;\ \ell_{n-1} \leftarrow \mathsf{read}$.)

Figures 6 and 7 show a variant of the previous program that uses these external functions with memory. The print_intr function now populates a buffer with the characters to be written and returns the length of the decimal representation of its argument (retval in the postcondition refers to the return value of the function), while print_int makes a single call to putchars with the populated buffer. The main function now reads four characters at a time and then processes them one by one, ultimately producing the same output as the previous program. The specifications for putchars and getchars describe changes to both external state and memory, as shown in Figure 5. Proving the specifications for the functions in this program is not any more difficult than in the memoryless case: we define an interaction tree main_loop capturing the slightly different pattern of interaction in this program, and then apply the appropriate separation logic rule to each command. The external calls affect both memory and the ITree predicate, while all other commands affect only memory and local variables, as usual.

[7] While these are not standard POSIX I/O functions, they are close to the behavior of POSIX `read`/`write`, socket operations, and other common forms of I/O.

$\{\mathsf{length}(\mathsf{decimal\_rep}'(i)) \leq \mathsf{length}(contents) \wedge$
$buf \mapsto contents\}$

```
int print_intr(unsigned int i,
      unsigned char *buf) {
  unsigned int q;
  unsigned char r;
  int k = 1;
  if (i!=0) {
    q=i/10u;
    r=i%10u;
    k = print_intr(q, buf);
    buf[k] = r+'0';
  }
  return k + 1;
}
```

$\{buf \mapsto contents[0...(\mathrm{retval} - 1) :=$
$\mathsf{decimal\_rep}'(i)]\}$

$\{\mathsf{ITree}(\mathsf{write\_list}(\mathsf{decimal\_rep}(i));;\ k)\}$

```
void print_int(unsigned int i) {
  unsigned char *buf = malloc(5);
  if (!buf) exit(1);
  int k;
  if (i==0){
    buf[0] = '0';
    buf[1] = '\n';
    k = 2;
  }
  else{
    k = print_intr(i, buf);
    buf[k] = '\n';
    k++;
  }
  putchars(buf, k);
  free(buf);
}
```

$\{\mathsf{ITree}(k)\}$

Fig. 6: A communicating program with memory (part 1)

## 5 Soundness of External-State Reasoning

The soundness proof of VST [1] describes the guarantees that the Hoare-logic proof of correctness for a C program provides about the actual execution of that program. A C program $P$ is represented as a list $P_1, ..., P_n$ of function definitions in CompCert Clight, a Coq representation of the abstract syntax of C. The program is annotated with a collection of function specifications (i.e., separation logic pre- and postconditions) $\Gamma = \Gamma_1, ..., \Gamma_n$, one for each function. We then prove that each $P_i$ satisfies its specification $\Gamma_i$, which we write as $\Gamma \vdash P_i : \Gamma_i$ (note that each function may call on the specification of any function, including itself). The soundness theorem of VST without external function calls is then:

**Theorem 1 (VST Soundness).** *Let $P$ be a program with specification $\Gamma$. Suppose for every function $P_i$ there is a proof $\Gamma \vdash P_i : \Gamma_i$ that $P_i$ satisfies its specification. Then the* `main` *function of $P$ can run according to the CompCert Clight semantics for any number of steps without getting stuck, and if it terminates then it does so in a state that satisfies its postcondition.*

*Proof.* First, make a nonstandard, ownership-annotated, resource-annotated, step-indexed small-step semantics for Clight. Define Verifiable C's Hoare triple as a shallowly embedded statement about safe executions in this "juicy" semantics. Then show that executions in the juicy semantics *erase* to corresponding safe executions in Clight's standard "dry" small-step semantics.

$\{\mathsf{ITree}(cs \leftarrow \mathsf{read\_list}(4);;\ \mathsf{main\_loop}'(0, cs))\}$

```
int main(void) {
  unsigned int n, d; unsigned char c;
  unsigned char *buf;
  int i, j; n=0;
  buf = malloc(4);
  if (!buf) exit(1);
  i = getchars(buf, 4);
  while (n<1000) {
    for(j = 0; j < i; j++){
      c = buf[j];
      d = ((unsigned)c)-(unsigned)'0';
      if (d>=10) { free(buf); return 0; }
      n+=d;
      print_int(n);
    }
    i = getchars(buf, 4);
  }
  free(buf);
  return 0;
}
```

$\{\mathsf{ITree}(\mathsf{done})\}$

Fig. 7: A communicating program with memory (part 2)

**Corollary 1.** *Since null pointer dereferences, integer overflows, etc. are all* stuck *in CompCert's small-step semantics, this means that a verified program will be free of all of these kinds of errors.*

This soundness theorem expresses the relationship between the juicy semantics described by VST's separation logic and the dry semantics under which C programs actually execute[8]. The proof of correctness of a program gives us enough information to construct a corresponding dry execution for each juicy execution[9]. However, we may not have access to the code of external functions, and in some cases (e.g., system calls) they may not even be implemented in C. In this section, we generalize the soundness theorem to include external functions.

[8] Of course, a C program *actually* executes by running machine code, but the relationship between the dry C semantics and the semantics of assembly language is already proved in CompCert, as is assembly-to-machine language [20].

[9] Theorem 1 blurs the line between juicy and dry by saying that a dry execution "terminates in a state that satisfies its postcondition", where the postcondition is stated in separation logic. In the original proof of soundness [1], this is resolved by assuming that the postcondition of `main` is always true. The techniques we use in this section can also be applied to more refined specifications of `main`.

In order to prove correctness of a C program with external calls in our separation logic, we must have a pre- and postcondition $\Gamma_i$ for each external function. At this level these specifications are taken as axioms, since we do not have access to the code of the external functions. To be able to describe the dry executions of programs that call these functions, we also need simpler specifications on dry states. Each *dry external specification* contains a pre- and postcondition for the function, which may refer to the memory state, arguments/return values, the external state, and a *witness* used to provide logical parameters to the pre- and postcondition. The core of our approach is to prove the correspondence between the juicy specification and the dry specification of each external function.

If we can relate every juicy specification to a dry specification, then why bother with the juicy specifications at all? The answer is, not every function can be specified "dry." Higher-order functions in object-oriented patterns, dynamically created locks with self-referential resource invariants, and many other C programming patterns cannot be given simple first-order specifications. But the *external functions* that correspond to ordinary input/output *can* be given first-order specifications. Therefore, users can write higher-order object-oriented programs, in which the *internal* functions have (only) juicy specifications, so long as the external functions have (also) dry specifications. For instance, consider the specification of the putchars function from the previous section:

$$\{\mathsf{length}(vs) = n \wedge \mathsf{ITree}(\mathsf{write\_list}(vs);;\ k) * buf \mapsto vs\}\ \text{putchars}(buf, n)$$
$$\{\mathsf{ITree}(k) * buf \mapsto vs\}$$

The pre- and postcondition each make one assertion about memory (that the buffer $buf$ points to the string of bytes $vs$) and one assertion about the external state[10] (that the interaction tree allows $\mathsf{write\_list}(vs)$ followed by $k$ before the call, and $k$ afterward). The corresponding first-order specification on dry memory and external state is:

$$\begin{aligned}\text{Pre}((vs,k),(buf,n),m,z) &\triangleq \mathsf{length}(vs) = n \wedge z = (\mathsf{write\_list}(vs);;\ k) \wedge \\ &\qquad \forall i < n.\ m(buf + i) = vs[i] \\ \text{Post}((vs,k),(buf,n),m_0,m,z) &\triangleq m_0 = m \wedge z = k\end{aligned}$$

where $(vs, k)$ is the witness (i.e., the parameters to the specification), $buf$ and $n$ are the arguments passed to the function, $m$ is the current memory, $z$ is the external state, and $m_0$ in the postcondition is the memory before the call (allowing us to state that memory is unchanged). Of the roughly 210 Linux system calls that are not Linux- or platform-specific, about 140 fall into this pattern, including socket, console, and file I/O, memory allocation, or are simpler informational calls like `gethostname` that do not involve memory.

Once we have a juicy and a dry specification for a given external function, what is the relationship between them? Intuitively, if the juicy specification for a function $f$ is $\{P_j\}\ f(args);\ \{Q_j\}$, the Hoare logic proof for a program that calls

[10] ITree is actually an assertion on the *external ghost state*, which is connected to the true external state as described in Section 3, and is erased at the dry level.

$f$ guarantees that $P_j$ is satisfied before every call to $f$, and relies on $Q_j$ holding after each such call returns. To know that the program will run without getting stuck, on the other hand, we must know that the dry precondition $P_d$ is satisfied before each call, and we can assume that the dry postcondition $Q_d$ is satisfied after each return. So informally, we need to know that $P_j$ implies $P_d$ and that $Q_d$ implies $Q_j$. This cannot be a simple logical implication, however, because $P_j$ and $Q_j$ are predicates on juicy memories, while $P_d$ and $Q_d$ are predicates on dry memories. A juicy memory $jm$ is a dependent triple $(m, \phi, pf)$, where $m$ is a dry memory, $\phi$ is a higher-order, step-indexed memory with ghost state, and $pf$ is a proof of the relationship between $m$ and $\phi$. We can easily extract the dry memory $m$ from a juicy memory (we write this as $\mathsf{dry}(jm)$), but there are many possible $\phi$'s that may correspond to a single $m$: we need to make decisions about ownership information and ghost state that is not present at the CompCert level.

In order to relate the juicy and dry specifications, we must erase the juice from the precondition, $P_j \Rightarrow P_d$, and then reconstruct the juice in the postcondition, $Q_d \Rightarrow Q_j$. The key to this erasure is that, as explained above, the $P_j$ and $Q_j$ for external functions generally make only first-order assertions on memory (memory buffers passed to system calls don't contain higher-order objects such as function pointers and locks). The rest of the memory is implicitly the *frame*, and will not be changed by the external call. For first-order predicates, erasure is injective, and the associated juicy memory can be uniquely reconstructed once the buffer has been modified. The frame *can* contain noninjective juice, but we can reuse the same juice in going from $Q_d \Rightarrow Q_j$ that we erased in going from $P_j \Rightarrow P_d$, since the external function does not modify the frame. In practice, the story is not quite so simple: the external function might allocate or free memory, the dry witness (used in $P_d$ and $Q_d$) must be derived from the juicy witness (used in $P_j$ and $Q_j$), and so on. We now formalize the details, culminating in Definition 6, the formal correspondence between juicy and dry specifications.

First, we address the problem of reconstructing a juicy memory from a dry memory. While there are many juicy memories that correspond to a given CompCert memory, it is easy to start with a (precondition) juicy memory and change it to reflect (postcondition) modifications to the associated dry memory, as long as those changes fall within certain limits. In particular, a memory location may be newly allocated or deallocated, or its value may be changed while staying at the same permission level, but its permissions should not otherwise be changed[11]. If a dry specification ensures that memory is changed in only (at most) these ways, we say that it *safely evolves* memory. When a user adds a new set of external functions to VST, this safe evolution property will be one of their proof obligations. As long as an external function satisfies a specification that safely evolves memory, we can always reconstruct the juicy memory after the call by modifying the original juicy memory to reflect the changes to the dry memory. This

[11] Any function that interacts with memory through the standard interface of load, store, alloc, and free will fall within these limits; concurrency operations, such as acquiring or releasing a lock, may not, and proving that lock operations are correctly implemented is outside the scope of this work.

reconstruction captures the effects of the external call on the program's memory; to reflect the changes to the external state, we must also set the external ghost state of the reconstructed juicy memory to match the external state returned by the call. We define a reconstruct operation such that $\mathsf{reconstruct}(jm, m, z)$ is a version of the juicy memory $jm$ that has been modified to take into account the changes in the dry memory $m$ and the external state $z$.

Second, we need a way to transform a juicy witness into the corresponding dry witness. When a user adds a new external call to VST, they must provide a dessicate function that performs this transformation. Fortunately, the dessicate operation usually follows a simple pattern. Components of the witness that are not memory objects are generally identical in their juicy and dry versions. The frame is usually the only memory object in the juicy witness; while it is possible in VST to write a Hoare triple that quantifies over other memory objects explicitly, it is very unusual and runs counter to the spirit of separation logic. Similarly, the postcondition of the dry specification may refer to the memory state before the call (to express properties such as "this call stored value $v$ at location $\ell$"), but there is rarely a reason to refer to any other memory object. Thus, the dessicate operation for each function can simply discard the frame (juicy) memory and replace it with the dry memory from before the call. This standard dessicate operation works for all external functions shown in this paper.

This leads to the following definition and theorem:

**Definition 6 (Juicy-Dry Correspondence).** *A juicy specification* $(P_j, Q_j)$ *and a dry specification* $(P_d, Q_d)$ *for an external function* correspond *if, for a suitable* dessicate *operation:*

- *for all witnesses $w$, arguments $a$, external states $z$, and juicy memories $jm$, if $P_j(w, a, z, jm)$, then $P_d(\mathsf{dessicate}(jm, w), a, z, \mathsf{dry}(jm))$; and*
- *for all witnesses $w$, arguments $a$, return values $r$, external states $z$, initial juicy memories $jm_0$, initial external states $z_0$, and dry memories $m$, if $P_d(\mathsf{dessicate}(jm_0, w), a, z_0, \mathsf{dry}(jm_0))$ and $Q_d(\mathsf{dessicate}(jm_0, w), r, z, m)$, then $Q_j(w, r, z, \mathsf{reconstruct}(jm_0, m, z))$.*

**Theorem 2 (VST Soundness with External Functions).** *Let $P$ be a program with $n$ functions, calling also upon $m$ external functions. The internal functions have (juicy) specifications $\Gamma_1 \ldots \Gamma_n$ and the external functions have (juicy) specifications $\Gamma_{n+1} \ldots \Gamma_{n+m}$. Suppose $P$ is proved correct in Verifiable C—there is a derivation $\Gamma \vdash P_1 : \Gamma_1, \ldots, P_n : \Gamma_n$. Let $D_{n+1}, \ldots, D_{n+m}$ be dry specifications that safely evolve memory, and that correspond to $\Gamma_{n+1} \ldots \Gamma_{n+m}$. Then the* `main` *function of $P$ can run according to the CompCert C semantics, using $D$ as the semantics of external function calls, for any number of steps without getting stuck, and if it terminates then it satisfies its postcondition.*

*Proof.* We extend the juicy semantics of Theorem 1 with a rule for external calls that uses their juicy pre- and postconditions, and then prove that executions in this semantics erase to safe executions in the dry semantics, using the correspondence to relate juicy and dry behaviors of external calls.

Although this theorem does not explicitly mention external communication, it implies that any I/O operations performed by $P$ conform to the description of allowed communication in the specification of `main`. This follows from the fact that only external calls can change the external state, and only external calls can communicate with the outside world. Thus, if $P$ performs a sequence of external function calls $f_1, ..., f_n$, the external communication performed by $P$ must be consistent with the specifications $D_{f_1}, ..., D_{f_n}$. In the case of the examples above, this means that at any point in a program's execution, its communication so far will be a prefix of the operations allowed by the initial ITree predicate, as desired.

Proving the correspondence between the juicy and dry specifications is the primary proof burden for a VST user who wants to use a new external function in their program. Fortunately, this proof only needs to be done once per external function rather than once per program (as long as the original specification is general enough to be usable in many different programs), and soundness (Theorem 2) has been proved once and for all. As a result, a VST user can prove that their program with external calls runs correctly as follows:

1. For each external function used in the program (that has not already been specified in VST), write a separation logic specification for that function.
2. Prove correctness of the program in VST as usual using the separation-logic-level external specifications.
3. For each external function used in the program (again, that has not already been specified), write a dry specification describing its effects on CompCert memories, and prove that the dry specification corresponds to the juicy specification and safely evolves memory.
4. Show immediately that the program runs correctly for any number of steps by applying Theorem 2.

For instance, we have already seen the VST-level specifications for putchars and getchars, and used them to prove correctness of a simple program; we can complete the process with the following lemma.

**Lemma 1.** *The juicy specifications of putchars and getchars correspond to their dry specifications.*

As a result, we now know that the sample program in Figure 7 runs correctly for any implementation of putchars and getchars that satisfy their dry specifications.

## 6 Connecting VST to CertiKOS

In the previous section, we showed how to connect a step-indexed separation logic specification of an external function to a "dry" specification on non-step-indexed CompCert memories and external state. This gives us a correctness property for C programs with external functions, but it still treats the dry specifications of the external functions as axioms. In this section, we show how to discharge these axioms by connecting dry specifications to implementations of the corresponding functions in the verified operating system CertiKOS [7].

```
Definition serial_in (port : Z) (st : OSState) : OSState * Z :=
  ... (* read buffers, compare bits, etc *)
  let new := st.(serial_oracle) st.(serial_trace) in
  match new with
  | SerialRecv data ⇒
    let (st', byte) := ... in (* manipulate data *)
    (st'/[serial_trace := st.(serial_trace) ++ [new]], byte)
  | ... (* handle other events *) end.
```

Fig. 8: A specification of a serial driver

### 6.1 CertiKOS Specifications

In order to explain how to connect VST and CertiKOS specifications, we first summarize how their specification styles differ. In VST, a specification is a pre- and postcondition on the (step-indexed, ghost-state-augmented) memory state of a program. In CertiKOS, a specification is a function representing a state transition from the current OS state to a new one with an (optional) return value. The OS state is a record with fields for each piece of concrete or logical state that CertiKOS maintains, such as page table maps and console buffers. Specifications are organized into "Certified Abstraction Layers" [6], which can be independently proven to refine higher-level abstractions, and then composed with other layers to build more complex systems. The concrete CertiKOS kernel implementation, in C and assembly, is verified with respect to high-level specifications using this layer framework and the CompCert compiler.

Because the specifications are pure, deterministic functions, something more is needed to model functions with externally visible effects such as I/O. To handle such functions, CertiKOS parameterizes specifications by "environment contexts" [8], which act as oracles that take a log of the events up to that point and return the next steps taken by the environment. Each oracle has a fixed set of events it can produce, along with a trace well-formedness invariant that it must preserve. For example, the oracle for modeling the behavior of the serial device can return events indicating the successful completion of a send or the arrival of some data, and it is assumed to only receive values that fit in a byte ([0, 255]). Although any particular choice of oracle is a deterministic function, its implementation is completely opaque to the specification, so that proofs about the specification's behavior hold given any oracle and environment state.

As a concrete example, consider the abridged specification of part of the serial driver in CertiKOS (Figure 8). After some initial work, the specification needs to know what bits came in from the physical device, so it consults the oracle and branches based on the next serial event. If the next event is a receive, it manipulates the received data to extract a byte and returns it along with a new state in which the trace is updated to include the processed event.

### 6.2 Relating OS and User State

```
Definition serial_putc (c : Z) (st : OSState) : option (OSState * Z) :=
  let c' := c mod 256 in
  if st.(ikern) && st.(init) && st.(ihost) then
    if st.(drv_serial).(serial_exists) then
      match st.(com1) with
      | mkDevData (mkSerialState _ true _ _ txbuf nil false) _ ltx _ ⇒
        let cs := if c' =? CHAR_LF then [CHAR_LF;CHAR_CR] else [c'] in
        Some (st/[com1/s/TxBuf := cs,
                  serial_log := st.(serial_log) ++ [IOEvPutc c]], c')
      | _ ⇒ None end
    else Some (st, -1)
  else None.
```

$$\mathrm{Pre}(k, c, m, z) \triangleq (\mathsf{write}(c);;\ k) \sqsubseteq z$$
$$\mathrm{Post}(k, c, m_0, m, z) \triangleq m_0 = m \wedge z \sqsubseteq k$$

Fig. 9: The core of the putchar system call vs. its dry specification

User-level programs cannot directly interact with the outside environment, and must instead communicate through the OS using the system call interface it provides. System calls in CertiKOS are specified just like any other operation, i.e., as a state transition function. For each system call, we would like to relate its dry pre- and postcondition (as described in Section 5) to its functional specification in CertiKOS. The property we would like to prove is something like: *for any initial state $s$, if the dry precondition holds for $s$, then the value $v$ and state $s'$ returned by the functional specification satisfy the dry postcondition.* Combined with the correspondence between juicy and dry specifications, this implies that the system call specification correctly implements the behavior expected by the user program (as expressed by its separation logic specification in VST). However, this property cannot be proven in its current form because the dry pre- and postconditions are predicates on CompCert memories and external state, which differ from CertiKOS's state, much of which is invisible and irrelevant to the user program, as can be seen in Figure 9. Instead, we must restate the correctness property in terms of relations between the common elements of the two state representations. The key components to relate are the return value of the system call, the representation of the user program's memory, and the model of external behaviors. The return value is a CompCert value in both systems, but the other two require additional work to translate between them.

Although, like VST, the CertiKOS kernel uses the CompCert C semantics and memory model, user-process memory is represented as a flat physical address space rather than a set of disjoint blocks. The OS state also includes page tables to map virtual to physical addresses and a record of which addresses are allocated. Fortunately, aside from these differences, the flat memory model is quite similar to CompCert's (see Figure 10). We assume the existence of a relation $R_{\mathrm{mem}}$ that maps blocks to virtual addresses. Other than the restriction

```
Inductive flatmem_val :=
| HUndef
| HByte: byte →flatmem_val.

(* Map from address to value *)
Definition flatmem :=
  ZMap.t flatmem_val.
```

```
Inductive memval :=
| Undef: memval
| Byte: byte →memval
| Pointer: block →int →nat →memval.
(* Map from block and offset to value *)
Record mem := mkmem {
  mem_contents: PMap.t (ZMap.t memval);
  ... }.
```

Fig. 10: A comparison of CertiKOS flat memory and CompCert memory that blocks fit in the virtual address space and map to nonoverlapping regions, the exact mapping has no effect on the system call correctness, so it can be completely arbitrary. To relate a CompCert memory to a CertiKOS one, we define a relation $\mathsf{inj}(m, \mathsf{flat}(s), \mathsf{ptbl}(s))$, which states that if a block and offset in the CompCert memory $m$ is valid, then it contains the same data as the corresponding location (according to $R_{\text{mem}}$ and the page table) in the flat memory of the OS state $s$. Note that $\mathsf{inj}$ is parameterized by the page table to allow a system call to alter the address mapping, for example by allocating new memory.

At the user level, the precondition contains an interaction tree (or similar external specification) that specifies the allowed external behaviors, and the postcondition contains a smaller tree that continues using the return value of the "consumed" actions. On the other hand, in CertiKOS, specifications begin with a trace of the events that have already happened and extend it with new events by querying the external environment. To reconcile these two views, we can first relate an interaction tree to a (possibly infinite) set of (possibly infinitely long) traces, each of which intuitively is the result of following one path in the tree. Then any trace allowed by the output interaction tree should be a suffix of a trace allowed by the input tree, and the difference between the two should be exactly the trace of events generated during the system call:

**Definition 7.** *We write* $\mathsf{consume}(\mathcal{T}, \mathcal{T}', tr)$ *to mean that, if* $tr'$ *is a trace of* $\mathcal{T}'$*, then* $tr \mathbin{++} tr'$ *(concatenation of* $tr$ *and* $tr'$*) is a trace of* $\mathcal{T}$*.*

Equipped with the relations defined above, we can define more precisely what it means for a system call to satisfy its dry specification.

**Definition 8 (Dry-Syscall Correspondence).** *A system call* $f$ *with functional specification* $O_f$ correctly implements *a dry specification* $(P_d, Q_d)$ *if for any arguments* $\boldsymbol{v}$*, CompCert memory* $m$*, interaction tree* $\mathcal{T}$*, and OS state* $s$*, if* $P_d(\boldsymbol{v}, m, \mathcal{T})$*,* $\mathsf{inj}(m, \mathsf{flat}(s), \mathsf{ptbl}(s))$*, and* $O_f(\boldsymbol{v}, s) = (s', v', t_{\text{new}})$*, then for all* $m'$ *such that* $\mathsf{inj}(m', \mathsf{flat}(s'), \mathsf{ptbl}(s'))$*, there exists* $\mathcal{T}'$ *such that* $\mathsf{consume}(\mathcal{T}, \mathcal{T}', t_{\text{new}})$*, and* $Q_d(\boldsymbol{v}, v', m', \mathcal{T}')$*.*

That is, if $f$ correctly implements a dry specification then for any state that satisfies the dry precondition $P_d$, we can inject the relevant piece of memory into an OS state $s$, apply the functional specification $O_f$, and then extract a resulting state that satisfies the dry postcondition $Q_d$. The inj relation may relate multiple CompCert memories to a given OS state (hence the universal quantification over the resulting memory $m'$), but all such memories must agree on the contents of all valid addresses, so the postcondition will usually hold for all $m'$ if it holds for any $m'$.

**Theorem 3.** *Putchar and getchar in CertiKOS correctly implement their dry specifications.*

While this correspondence is specific to CertiKOS, we can adapt it to other verified operating systems by replacing the CertiKOS system call specification, user memory model, and external event representation with those of the other OS. For example, in the case of the seL4 microkernel [12], inj could be redefined to relate a CompCert memory to certain capability slots that represent the virtual memory, and the system call might send a message to a device driver running in another process. Despite these changes, most of the theorems in this paper aside from Theorem 3 would continue to hold with minor or no alterations.

### 6.3 Soundness of VST + CertiKOS

In Section 5, we described a correspondence between "juicy" separation logic specifications for external functions and "dry" CompCert-level specifications that is sufficient to guarantee that verified C programs behave correctly when run, as long as the external functions actually satisfy their dry specifications. Now we have seen how to prove that an external function satisfies its dry specification, by relating it to its CertiKOS specification. We combine these two proofs to get a stronger correctness property for programs that use CertiKOS system calls. This will also allow us to formalize the idea that at each point in a program's execution, it has performed some prefix of the communication operations specified in its precondition.

First, we define the semantics of programs with respect to the implementation of external functions:

**Definition 9 (OS Safety).** *Suppose that we have a set of external calls $F$ such that each $f \in F$ has a functional specification $O_f$. Then a configuration $(c, m, t, \mathcal{T})$, where $c$ is a C program state, $m$ is a memory, $t$ is a trace of events performed so far, and $\mathcal{T}$ is an interaction tree specifying the allowed future events, is* safe for $n$ steps with respect to a set of traces $T$ *if:*

- *$n$ is 0 and $T$ is $\{\epsilon\}$, or*
- *$(c, m) \to (c', m')$ and $(c', m', t, \mathcal{T})$ is safe for $n - 1$ steps with respect to $T$, or*
- *$c$ is at a call to an external function $f$ with arguments $\boldsymbol{v}$, and for all $s$ consistent with $t$ such that $\mathsf{inj}(m, \mathsf{flat}(s), \mathsf{ptbl}(s))$, if $O_f(\boldsymbol{v}, s) = (s', v', t_{\text{new}})$, then there is some new interaction tree $\mathcal{T}'$ such that $(c', m', t \mathbin{++} t_{\text{new}}, \mathcal{T}')$ is safe for $n - 1$ steps with respect to $T'$, where $c'$ is the program state after the call (using the return value $v'$), $\mathsf{inj}(m', \mathsf{flat}(s'), \mathsf{ptbl}(s'))$, and $\mathsf{consume}(\mathcal{T}, \mathcal{T}', t_{\text{new}})$, and $T$ is the union of $\{t_{\text{new}} \mathbin{++} t' \mid t' \in T'\}$ for all such $T'$.*

The C program has states $(c, m)$, where $c$ holds the values of local variables and the control stack, and $m$ is the memory. Our small-step relation $(c, m) \rightarrow (c', m')$ characterizes *internal* C execution, and therefore if $c$ is at a call to an external function then $(c, m) \not\rightarrow (c', m')$. The operating system has states $s$ that contain the physical memory $\mathsf{flat}(\mathsf{s})$ and many other components used internally by the OS (and its proof of correctness), including a trace of past events; we say that $s$ is consistent with $t$ when the trace in $s$ is exactly $t$.

Definition 9 has several important differences from our original definition of safety in Section 2. First, configurations include the trace $t$ of events performed so far, as well as $\mathcal{T}$, the high-level specification of the allowed communication events (here it is taken to be an interaction tree, but it could easily be defined in another formalism just by changing the definition of $\mathsf{consume}$). Second, our external functions are not simply axiomatized with pre- and postconditions, but implemented by the executable specifications $O_f$ provided by the operating system. We use the ideas of the previous section to relate the execution of C programs to the behavior of system calls: we inject the user memory into the OS state, extract the resulting memory from the resulting state, and require that the new interaction tree $\mathcal{T}'$ reflect the communication events $t_{\text{new}}$ performed by the call. Note the quantification over the current OS state $s$: the details of the OS state, such as the buffer of values received, are unknown to the C program (and may change arbitrarily between steps, for instance, if an interrupt occurs), and so it must be safe under all possible OS states consistent with the events $t$. The set $T$ contains all possible communication traces from the program's execution, so by proving that every trace in $T$ is allowed by the initial interaction tree $\mathcal{T}$, we show that the program's communication is always constrained by $\mathcal{T}$.

**Lemma 2 (Trace Correctness).** *If $(c, m, \mathcal{T})$ is safe for $n$ steps with respect to $T$, then for all traces $t \in T$, there exists some interaction tree $\mathcal{T}'$ such that $\mathsf{consume}(\mathcal{T}, \mathcal{T}', t)$.*

*Proof.* By induction on $n$. Since the $\mathsf{consume}$ relation holds for the trace segment produced by each external call, it suffices to show that it is transitive, i.e., that $\mathsf{consume}(a, b, t_1)$ and $\mathsf{consume}(b, c, t_2)$ imply $\mathsf{consume}(a, c, t_1 +\!\!+\, t_2)$.

**Theorem 4 (Soundness of VST + CertiKOS).** *Let $P$ be a program with $n$ functions, calling also upon $m$ external functions. The internal functions have (juicy) specifications $\Gamma_1 \ldots \Gamma_n$ and the external functions have (juicy) specifications $\Gamma_{n+1} \ldots \Gamma_{n+m}$. Suppose $P$ is proved correct in Verifiable C with initial interaction tree $\mathcal{T}$. Let $D_{n+1}, \ldots, D_{n+m}$ be dry specifications that safely evolve memory, and that correspond to $\Gamma_{n+1} \ldots \Gamma_{n+m}$. Further, let each $D_i$ be correctly implemented by an OS function $f_i$ with executable specification $O_{f_i}$. Then for all $n$, the `main` function of $P$ is safe for $n$ steps with respect to some set of traces $T$, and for every trace $t \in T$, there exists some interaction tree $\mathcal{T}'$ such that $\mathsf{consume}(\mathcal{T}, \mathcal{T}', t)$.*

*Proof.* By the combination of the soundness of VST with external functions (Theorem 2), Lemma 2, and a proof relating our previous definition of safety to the new definition.

This is our main result: by combining the results of the previous sections, we obtain a soundness theorem down to the operating system's implementation of system calls, one that guarantees that the actual communication operations performed by the program are always a prefix of the initial specification of allowed operations. By instantiating the theorem with a set of verified system calls, we obtain a strong correctness result for our VST-verified programs, such as:

**Theorem 5.** *Let $P$ be a program that uses the putchar and getchar system calls provided by CertiKOS, such as the one in Figure 4. Suppose $P$ is proved correct with initial interaction tree $\mathcal{T}$. Then for all $n$, the* `main` *function of $P$ is safe for $n$ steps with respect to some set of traces $T$, and for every trace $t \in T$, there exists some interaction tree $\mathcal{T}'$ such that* $\mathsf{consume}(\mathcal{T}, \mathcal{T}', t)$.

## 7 From syscall-level to hardware-level interactions

Thus far, we have assumed that the events in a program's trace are exactly the events described in the user-level interaction tree $\mathcal{T}$. In practice, however, the communication performed by the OS may differ from that observed by the user. For example, like all operating systems, CertiKOS uses a kernel buffer of finite size to store characters received from the serial device; if the buffer is full, incoming characters are discarded without being read. To represent this distinction, we distinguish between the user-visible events produced by system calls, and *external events*, which are generated by the environment oracle and recorded in the trace at the time that they occur. For the system call events to be meaningful, they must correspond in some way to the external events, but this correspondence may not be one-to-one. In the case of console I/O, each character received by the serial device should be returned by getchar at most once, and in the order they arrived, but characters may be dropped. This leads us to the condition that the user events should be a subsequence of the environment events, which is proved in CertiKOS.

**Lemma 3.** *The getchar system call maintains the invariant that there exists an injective map from a system call event with value $v$ in the OS trace to an external event with value $v$ earlier in the trace.*

**Corollary 2.** *Let $P$ be a verified program as described in Theorem 4, in which getchar is the only system call performed. Then for all $n$, the* `main` *function of $P$ is safe for $n$ steps with respect to some set of traces $T$, and for every trace $t \in T$, there exists some interaction tree $\mathcal{T}'$ such that* $\mathsf{consume}(\mathcal{T}, \mathcal{T}', t)$*, and the events in $t$ correspond to external events performed as described in Lemma 3.*

Unlike Theorem 4, this corollary is specific to a particular system call, but it gives a stronger correctness property: the events in the user-level interaction tree are now interpreted in terms of actual bytes received by the OS, in the form of external events. Note that Lemma 3 does not require that every external event has a corresponding system call event; if the buffer fills up and characters are dropped before a getchar call, then there will be external events that do not correspond to anything in the interaction tree, and this is the intended semantics of buffered communication without flow control. A similar corollary can be proved for any set of system calls, but the precise correspondence between user events and external events will depend on the particular system calls involved.

There is one more soundness theorem we might want to prove, asserting that the combined system of program and operating system executes correctly according to the assembly-level semantics of the OS. We should be able to obtain this theorem by connecting Theorem 4 with the soundness theorem of CertiKOS, which guarantees that the behavior of the operating system running a program $P$ refines the behavior of a system $K \bowtie P$ consisting of the program along with an abstract model of the operating system. However, this connection is far from trivial: it involves lowering our soundness result from C to assembly (using the correctness theorem of CompCert), modeling the switch from user to kernel mode (including the semantics of the trap instruction), and considering the effects of other OS features on program behavior (e.g., context switching). We estimate that we have covered more than half of the distance between VST and CertiKOS with our current result, but there is still work to be done to complete the connection. We can now remove the OS's implementation of each system call from the trusted computing base; it remains to remove the OS entirely.

## 8 Related Work

The most comprehensive prior work connecting verified programs to the implementation of I/O operations is that of Férée et al. [5] in CakeML, a functional language with I/O connected to a verified compiler and verified hardware. As in our approach, the language is parameterized by functional specifications for external functions, backed by proofs at a lower level. However, while CakeML does support a separation logic [9], it is not higher-order, so all of the components are specified in the same basic style. Our approach could enable higher-order separation logic reasoning about CakeML programs. Ironclad Apps [10] also includes verified communicating code, for user-level networking applications running on the Verve operating system [21]. However, their network stack is implemented outside of the operating system, so proofs about I/O operations are carried out within the same framework as the programs that use the operations.

One major category of system calls is file I/O operations. The FSCQ file system [2] is verified using Crash Hoare Logic, a separation logic which accounts for possible crashes at any point in a program. File system assertions are similar to the ordinary points-to assertions of separation logic, but may persist through crashes while memory is reset. In Crash Hoare Logic, the implementation-level model of the file state is the same as the user's model, and the approach does not obviously generalize to other forms of external communication.

Another related area is the extension of separation logic to distributed systems, which necessarily involves reasoning about communication with external entities. The most closely related such logic is Aneris [14], which is built on

Iris, the inspiration for VST's approach to ghost state. The adequacy theorem of Aneris proves the connection between higher-order separation logic specifications of socket operations and a language that includes first-order operational semantics for those functions. In our approach, this would correspond to directly adding the "dry" specifications for each operation to the language semantics, and building the correspondence proof for those particular operations into the soundness theorem of the logic; our more generic style of soundness theorem would make it easier to plug in new external calls. The bottom half of our approach—showing that the language-level semantics of the operations are implemented by an OS such as CertiKOS—could be applied to Aneris more or less as is. Another interesting feature of Aneris is that the communication allowed on each socket is specified by a user-provided protocol, an arbitrary separation logic predicate on messages and resources. In our examples thus far, we have assumed that the external world does not share any notion of resource with the program, and so our external state only mentions the messages to be sent and received; however, the construction of Section 3 does allow the external state to have arbitrary ghost-state structure, which we could use to define similarly expressive protocols.

## 9 Conclusion and Future Work

We have now seen how to connect programs verified using higher-order separation logic to external functions provided by a first-order verified system, effectively importing the results of outside verification (e.g. OS verification) into our separation logic. The approach consists of two halves: we first relate separation logic specifications for the external functions to "dry" first-order specifications on CompCert memories [15] and interaction trees [13], and then relate these dry specifications to the system that implements the functions (CertiKOS in our example). In the process, we interpret the C-level communication constraints in terms of OS-level events that more accurately represent the communication that occurs in the real world. Our approach works for any type of external communication, and allows users to extend the system with new external functions as needed. Each new correspondence proof for an external function modularly extends the soundness theorem of VST, removing the separation-logic specification of the function from the trusted computing base.

The combination of CompCert memories with interaction trees has served as a robust specification interface between two quite different approaches to verification: VST's higher-order impredicative concurrent separation logic, and CertiKOS's certified concurrent abstraction layers. This strongly suggests that the combination of CompCert memories and interaction trees can serve as a *lingua franca* to interface with other verification systems for client programs and for operating systems.
